# Assessment of fall-associated risk factors in the Muslim community-dwelling older adults of Peshawar, Khyber Pakhtunkhwa, Pakistan

**DOI:** 10.1186/s12877-023-04322-1

**Published:** 2023-10-05

**Authors:** Rashida Bibi, Zhang Yan, Muhammad Ilyas, Mussarat Shaheen, Satya Narayan Singh, Akhter Zeb

**Affiliations:** 1 Institution of Nursing and Health Sciences, Zhengzhou University, Zhengzhou, Henan China; 2 School of Nursing, Iqra National University, Peshawar, Khyber Pakhtunkhwa Pakistan; 3 Government Nursing College Abbottabad, Khyber Pakhtunkhwa, Pakistan; 4 Torrens University, Adelaide, South Australia Australia; 5 Ismail College of Nursing Sawat, Khyber Pakhtunkhwa, Pakistan

**Keywords:** Falls, Risk factors, Community-dwelling, Older adults, Pakistan

## Abstract

**Background:**

Falls are the third-leading cause of disability among the elderly population worldwide. It is multifactorial, and the occurrence of falls depends on different factors, which can be different from context to context, and individual to individual. Therefore, regular assessment of fall risk factors is required to develop a strategy for fall prevention. The study aimed to identify fall-related risk factors in Pakistani healthy older adults at risk of developing physical disabilities. It also aimed to create a risk-predictive model for fall occurrence, offering evidence for preventive strategies.

**Methods:**

Data were collected from 140 Muslim older adults from two residential areas of Peshawar, Khyber Pakhtunkhwa, from July 2022 to August 25, 2022, after obtaining permission from the Zhengzhou University Ethical Review Board (ZZUIRB #202,254), and the District Health Department Office (DHO #14,207). Participants were informed, and consent was obtained before data collection. Data were collected using the Time Up and Go Test (TUGT) checklist, the Cognitive Screening Scores (CS-10) checklist, interviews regarding the prayer practice, fall history in the last six months, visual equity questions, and demographic variables.

**Results:**

Factors associated with falls were; age, gender, education, cognitive status, poor walking speed, lack of physical activity, poor vision, and history of falls in the last six months, with a significant *P* value of (*P*. < 0.05) in the Pearson correlation coefficient test. Poor cognition, low visual equity, poor walking speed, and lack of exercise increase the risk of falling in the future, with a prediction value of (*P* < 0.005) in Omnibus, Lemeshow score of (0.77).

**Conclusion:**

Hence, our study provides a road map for future risk assessment of falls by adding the four mentioned risk factors in the proposed model to facilitate timely action to prevent fall-related infirmities in Pakistani healthy older adults.

## Introduction

The aging population presents significant challenges to healthcare systems worldwide. The World Health Organization (WHO) defines individuals aged 60 years or older as part of the aging population [1]. The aging population poses significant challenges to healthcare systems globally, including in Pakistan, and the quality of life among older adults is not satisfactory [1]. Falls are a major cause of disability and prolonged hospitalization [2]. According to the Centers for Disease Control (CDC) report, worldwide, 36 million elders report falling each year, which accounts for nearly 33% of the prevalence of falls in community-based older adults [3]. Females over 70 are more likely to experience falls [4, 5]. The frequency of falls varies across countries. China reported a prevalence of 31 to 34%, Japan at 21%, and Latin America reported 16%, in Pakistan, approximately 44% of falls were documented in a survey study. Out of these falls, 8% resulted in injuries, placing individuals at a high risk for hospitalization or even premature death [2]. Therefore, older individuals in the Pakistani community should undergo regular fall risk assessments to develop a fall prevention strategy. With a projected increase in the number of individuals over 60 years old, it is crucial to understand the risk factors associated with falls in order to develop a healthy community. Therefore, this study aims to explore the prevalence of falls and associated factors in community-based older adults of Khyber Pakhtunkhwa, Pakistan, where the majority of the population belongs to the Muslim community. It is evident that Muslim communities have their own unique values and traditional systems. In these communities, females are often encouraged to stay at home while males are responsible for outdoor chores. However, this division of roles may increase the risk of falling for both genders.

Aging is characterized by a gradual deterioration in physiological and functional capacities. This includes the musculoskeletal and neurological systems, cognitive well-being, and sensory functions, as well as the onset of non-communicable diseases such as diabetes, cardiovascular diseases, and respiratory conditions. These factors collectively influence the activity levels of older adults and increase the likelihood of experiencing falls and disability [6, 7]. However, it is important to note that certain environmental factors also contribute to falls, such as slippery floors, inadequate lighting, and infrastructure that is not conducive to safe movement [8]. A comprehensive review study has also suggested that the risk of falls increases in advanced age due to a progressive decline in neurological and functional status [9].

The occurrence of falls in older adults is multifactorial. One out of four older adults aged 65 or older falls at least once a year. These falls are mostly contextual and individualized in nature. They occur when physical, psychological, social, and environmental factors work together to contribute to their prevalence [5, 10, 11].

Several studies have investigated the risk factors for falls, mostly reported from developed countries [7, 12]. he risk factors of falling can be divided into intrinsic factors, such as low energy levels, cardiovascular diseases, disability, and poor vision, and extrinsic factors such as housing conditions, economic status, and weather conditions etc. [6, 13]. Functional declines are increasing due to a lack of exercise, and physical activities, which increases the risk of falling, as reported in previous studies [4]. Exercise has emerged as a promising and evidence-based intervention to reduce the risk of falls among older adults. The body of evidence addressing how exercise helps prevent falls in older individuals is extensive and consistently supports the idea that exercise programs can significantly reduce the risk of falls. Together, strength training, balance and gait training, flexibility exercises, and aerobic activities, when combined, improve physical function, reduce the risk of fall-related injuries, and enhance overall quality of life. Customized exercise regimens that are tailored to individuals' abilities, skills, and preferences can assist older individuals in maintaining their independence, mobility, and overall well-being. This, in turn, can lead to a healthier and safer aging process, regardless of any existing health condition [14–16]. Physical activity level is primarily assessed by habitual walking speed [11, 15]. The Time Up and Go (TUG) is commonly used to assess functional health-related fall risk in older adults, whether they have physical mobility problems or not [17].

Several body movements, such as bowing and prostration, are therapeutic exercises that are similar to range of motion exercises [18]. These movements can improve visual and cognitive health by increasing blood circulation and sugar levels. Mild to moderate level of therapeutic exercises are essential to maintain cardiovascular fitness such as orthostatic hypotension, high blood pressure, and improving muscular strength, coordination, [7, 14, 19], prevent lumbar spondylosis, diabetes, and urinary incontinence (UI), highly correlated with falling history of older adults in previous studies [5, 15, 16, 20], and cause premature deaths [3, 4]. Falling does not solely depend on personal factors; there are also multiple external factors. Commonly identified home hazards that increase the risk of falls in older adults include difficulties with home access, such as navigating around the house, slippery floors, and falls specifically related to the bathroom. Previous studies have shown that these factors significantly contribute to the occurrence of falls [6, 11, 12]. Fear of falling, even without any fall history, is another factor in falls in older adults. This fear may restrict their activities, and ultimately, they may adopt a sedentary lifestyle [21]. According to Cameron and others, contain central nervous system depressant drugs, such as antihypertensive, can lower blood pressure and impair cognitive function, leading to blurred vision and postural hypotension [6, 22]. Oshiro et al. conducted a study to predict the risk of falling by considering a combination of psychological and medical characteristics, medication usage, and sensory factors [23]. The study found a significant 98% positive correlation between adjusted variables and fall risk, as well as an 8% negative correlation. A cross-sectional study reported that falls were higher among sedentary older adults compared to those who engaged in daily basis [2]. Another study mentioned that prescribing appropriate body movements during Muslim prayers holds substantial promise in preventing falls in older adults. Regular performance of these movements can improve musculoskeletal strength, balance in gait, cardiovascular and respiratory system health, and metabolism [24]. Older adults in developing countries face a higher risk of sustaining fall-related injuries [25]. This can be attributed to several factors, including non-conducive environmental conditions, limited access to health facilities and screening opportunities, lack of awareness regarding healthy aging, lack of exercise, and lack of social support [12, 26]. Identifying and addressing fall-related risk factors enables healthcare professionals to establish safer home environments and manage the modifiable risk factors associated with falls in older adults.

Identifying older adults who are at higher risk of falling and require interventions is challenging for clinicians and public health professionals. However, various fall predictors and scales have been developed and tested in various settings to assess fall risk among older adults. The Time Up and Go test (TUG) is the most frequently used tool to assess fall risk in older adults. It is assessed by having the participant rise from a chair, walk three meters, turn around, walk back, and sit down on the chair within an assigned time. The systematic review study mentioned that the TUGT score varies for different age groups and depends on the individual's health condition. Another review study claimed that the threshold time demarcating individuals classified as non-fallers and fallers exhibited variation, ranging from 10 s to 32.6 s [27]. Furthermore, an average time of less than 19 s for a 3m walk can be considered normal functional mobility for a healthy older adult [28]. Lusardi et al. included the Mini-Mental State Examination (MMSE) in their predictive model [29]. Another study mentioned a comprehensive list of risk indicators, emphasizing the high predictive value of variables such as TUG, visual acuity, and cognitive scores [17]. Moreover, a 12-point checklist consisting of medical history, medication use, balance, gait, muscle strength, and environmental hazards is frequently used in clinical settings to assess fall risk among older adults [7]. Due to its multi-factorial nature, the researchers focus on formulating predictive models to assess fall related risk factors based on the individualized and environmental determinants that can be different from context to context. Therefore, regular fall risk assessment is crucial to preventing fall-related injuries in older adults living in developing countries [30], where they are more susceptible to fall-related disabilities [6, 29].

Within the scope of fall risk assessment and its multifactorial nature, the current challenge lies in the inability to directly apply risk factor assessment checklists to a specific demographic and environmental context. The debate in the literature revolves around the possibility of predicting falls through various models available to assess fall risk in older adults without any neurological decline and includes interventions that contribute to fall prevention [31]. A systematic study was conducted to examine all prognostic predictive models for fall risk assessment, which included 30 papers on fall risk predictions. The results supported that a history of falls, age over 75, being female, having TUG scores > 19, poor vision, and a level of disability cutoff point > 5 were predictors of fall occurrence in older adults [6]. The collective accuracy of these predictions were ranged from 0.62 to 0.69 in the previous study [23], indicating inconsistency in the predictive factors of falls.

There are several predictive models and fall assessment checklists available, but they may not be effective in preventing falls in a population with different geographic and demographic characteristics. Although estimating gait quality scores with TUGT cut-off points, cognitive function status, past incidence of falls, and visual health may be common factors among individuals above 60 years of age group [31]. Mishra and colleagues conducted a study in the United States of America (USA) and developed a predictive model. The cutoff point for "time-up and go" test scores was determined to be > 12 s for a 3 m walk. Additionally, they used a cognitive screening (10-CS) scale with a cutoff point of < 7 and considered individuals with a history of at least one fall in the last six months. The model was highly sensitive with an Area Under Curve (AUC) of 0.80 (95% CI 0.76–0.85), and a sensitivity of 0.82 (95% CI 0.74–0.89) [32]. These parameters have been utilized in multiple falls risk assessment studies as a standard checklist for community-based older adults without any neurological health declines.

Diverse methods have been used in the literature to develop fall risk assessment models, including decision tree analysis [27], fall prediction algorithms [28], logistic regression [31], and receiver operator characteristic (ROC) analysis for fall risk screening in older adults [28]. In the field of predictive models, the six key predictors have shown the ability to accurately predict fall events, with probabilities ranging from 30.4% to 71.9%. A comparative review of predictive models measured parameters, such as area under the curve of any predictive factors (0.70 vs. 0.64), accuracy (0.65 vs. 0.62), sensitivity (0.62 vs. 0.50), positive predictive value (0.66 vs. 0.65), and negative predictive value (0.66 vs. 0.65). These parameters can be used as standard measures for fall risk assessment.

It is important to develop contextual and individualized intervention strategies to prevent falls in older adults from different populations. To achieve this, an updated assessment checklist is needed to evaluate the risk of falls in Muslim older adults living worldwide.

There is a noticeable gap in the research, as many studies have mentioned the beneficial effects of exercise and physical activities in preventing falls in older adults. However, Muslim people claim to perform five-time prayers with seven distinct body movements [29], they argue that these scientifically based therapeutic exercises help keep joints, bones, and muscles healthy, improve cognition, enhance sleep quality, promote blood circulation at the capillary level, and reduce pain [24, 33]. According to my knowledge, no study has been conducted to evaluate the beneficial effect of the seven steps of prayers in preventing falls among older Muslim adults, except for a few studies carried out to assess the actual level of performance in different Muslim countries such as Malaysia and Iran [24, 34]. Older adults in the Muslim community primarily prioritize prayers. However, the quality of life and fall prevalence do not differ significantly from other communities. Therefore, an objective assessment is needed to evaluate fall risk. This assessment will involve assigning scores to seven steps, ranging from 1 to 7. A cutoff point of 5 scores will be used to determine the validity of the checklist in relation to fall risk. Incorporating statistical techniques, such as binary regression models, holds the potential to reveal predictive capacities and identify threshold values. This can help determine if non-compliance with the seven stages correlates with a higher risk of falls [35]. Therefore, the purpose of our study was to identify potential contextual risk factors associated with falls in Muslim adults who were at risk for falling. Our goal was not only to identify risk factors for falls but also to develop a predictive model that highlights the intricate relationship between the lack of body movement during Muslim prayers and the risk of falls in older adults.

## Materials and methods

### Design and setting

The data was collected from the baseline data of an interventional study conducted in two residential areas of Peshawar district, Khyber-Pakhtunkhwa, Pakistan, from July 2022 to August 2022, from the 140 study participants. The sample size was calculated using G-power, assuming a significance level of 0.05 (α), a power of test of 0.8 (β), and a confidence level of 95% [36].

### Inclusion and exclusion criteria

The inclusion criteria for this study included older adults aged 60 or older, living in the community, without a clinical diagnosis of a neurological or musculoskeletal disorder such as Parkinson's disease or arthritis, and reporting less than 2 h of daily physical activity. Those participants who are able to follow directions, have no severe memory problems (dementia), and can walk independently without assistance. In contrast, participants were excluded if they had language challenges, showed a lack of interest in participating, or were actively engaged in paid or unpaid volunteer work [37].

### Ethical considerations

The study adhered to the principles outlined in the Helsinki Declaration. Permission was obtained from the parent institution, Zhengzhou University, Institute of Ethical Review Board (ZZUIERB #202,254), and the District Health Department Office (DHO #14,207). The participants were informed about the study objectives, their voluntary participation, and their ability to withdraw from the study at any point. Written informed consent was obtained from all study participants. Anonymity was maintained, data was secured, and it was not disclosed to others at any point during the study.

### Data collection process

Data were collected using validated [1] and reliable checklists such as, the 10-point Cognitive Screening (10-CS) scale [28], Time Up and Go (TUG) [20], age categories, and visual health assessments used in previous studies [5, 17, 38]. Moreover, a scale of seven steps of Muslim prayer, ranging from 1 to 7, was included to predict the risk of falls in community-based older adults.

#### Physical test

**Fig. 1:**
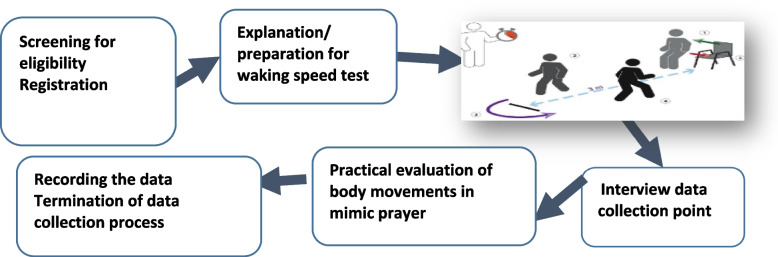
Step by step process for falls risk assessment of study participants = 140

The Time Up and Go test was performed in three primary healthcare units (BHUs) in Peshawar. A trained physiotherapist conducted the timed "Up and Go" test (TUG), in which scores were assigned based on the time taken to go and sit down. A time of 10 to 19 s was considered normal, 20 to 29 s was included in the gray list indicating fall risk, and a time of > 30 s was considered highly risky for falling [29]. The time it took for participants to rise from a chair, walk three meters, turn around, walk back, and sit down on the chair was recorded. During the test, individuals are instructed to wear their regular, comfortable footwear and use any mobility aids (e.g., a cane or walker) that they would normally require. The process is highlighted in Fig. 1.

#### Cognitive status examination

The 10-point Cognitive Screener (10-CS) was developed by the National Institute of Health and Care Excellence to assess the current cognitive level of highly educated older adults. This tool was consistent with our population, in which more than 75% of older adults are illiterate. The tool consists of three sequential orientation questions (year, month, and date), a three-word recall, and a four-point scaled animal naming task. Scores ranging from 0 to 1 are allocated for each item, and for the animal naming task, the scores range from 0 to 5. A score of zero is given for naming 0–5 animals, while 4 points are awarded for naming more than 15 animal names. A score of 8 or higher is considered normal, while a score of 6–7 suggests possible cognitive impairment. A score of 0–5 indicates probable cognitive impairment [39].

#### Body movements and physical activity

The participants' physical activity was assessed by asking about the seven body movements they perform during their prayers. Revised 2: There are seven steps in the Muslim prayer. These steps include: 1) Takbir: The prayer sequence begins with raising hands and moving them to the face level. This movement stimulates joints, facial muscles, and cervical muscles. 2) Standing: This step lasts for 60–90 s, with arms wrapped in front of the belly. It activates scapular retractors, stretches pectoral muscles, and activates deep neck flexors. 3) Rukuk: This step involves a forward bending position at the thoracic and lumbar spines. It stretches various muscles and structures and resembles a slump position, stretching posterior musculature and dura. 4) Qiam: After Rukuk, there is a 5-s period of standing. During this time, the chin is tucked in, eyes are pointing in one place, and it stimulates eye vision and neck muscles. 5) Prostration: Moving from standing to prostration involves flexion in the thoracic and lumbar spines, hip flexion, and mid-flexion in the knees. This posture improves muscles such as the biceps brachii, triceps brachii, and pectoralis major. Sitting (Jalsa): In a sitting position, the lumbar and thoracic spines are neutral, with the hip and knee joints in flexion. This exercise stretches the quadriceps femoris muscle. Additionally, turning the head to both shoulders in a rhythmic motion improves eye movement and strengthens the cervical muscles. These steps are similar to meditative postures, such as those in yoga, and they offer physical fitness benefits as well as overall improvements to bodily systems.

We considered a cutoff point of 5 correctly performed steps scores, where > 5 correct answers indicated the correct performance of prayers in this study. The same questions were mentioned in the previous study [33]. The following questions were included in the assessment of prayer performance checklist:2) During the prayer, can you stand easily and stabilize your body as part of the prayer sequence?;3) Does your daily prayer involve a bowing movement known as Rukuk in the prescribed manner?4) Do you perform the five-time prayer in the traditionally prescribed manner? Do you perform prostration (sujood) without any pressure on your head? Do you easily bend both of your legs during Jalsa without any difficulty or pain? Do you move your head completely from side to side without any difficulty during Salam?

### Statistical analyses

Using the Statistical Package for the Social Sciences (SPSS version 23). Descriptive statistics and the Spearman correlation coefficient test were used to describe and analyze factors related to the occurrence of falls in the last year at a significance level of (*P* < 0.05). A binary logistic regression model was used to test the following hypotheses of this study, which were: 1) H1. Fall occurrence is higher in those with low-speed in walking; 2) H2. The falling ratio is high with regard to visual and cognitive problems. The data were checked for normality. The feature significance values for each feature were assigned a distribution. For instance, lower gait speed values, poor prayer postures, and poor cognition scores were hypothetically negatively correlated with fall risk.

The area under the curve (AUC) was measured to check the sensitivity and specificity of the model under study, and if we kept > 75% of the test, it will be considered valid and reliable to check the fall's occurrence with the selected parameters [28].

#### Model fit index

In this model, we considered total scores = 0–1 in which 1 indicated fall risk and 0 indicated no falls risk. Our preferred choice is score = 1 because we are interested in seeing the drops in respondents. A score of zero value is given a benchmark, as it is significant at 0.03 and shows that when we add an independent variable (the risk factors), it will predict the dependent variable that is fall risk in our model. We applied the Omnibus model of coefficient testing considering the difference between the baseline model and the new model, the Nagelkerke (R2) square > 0.50, the pseudo-R square to check the approximate variation in the criterion variable were adjusted for Cox and Snell R, that is, Nagelkerke R2 > 0.50 in the criterion variable can be accounted for in the predictive variable. Receiver operating characteristic (ROC) curves and logit rank plots were plotted for each variable in the models to help determine the list of critical measures identified through binary regression modeling.

#### Sensitivity and specificity analysis

Using ROC the standard errors for the areas un-der the ROC curve were determined in SPSS, version 23. For each sample participant, the projected probability—calculated as elogit (p)/(1 + elogit(p)), where logit(p) is the indicator derived from the regression model was also analyzed. The specificity of the model was assumed that the Receiver Operator Characteristic (ROC) with > 75% AUC accurately measures the association of independent variables such as low physical activity, poor vision and cognition on fall risk. We considered that the model correctly predicted not to fall risk, and the model sensitivity ROC > 75% was assuming that the model correctly predicting to fall risk is due to the selected variables [32]. The cut off point for TUG was =  < 19 s, cognitive function =  < 5 scores, prayer method = 5, and visual function = 1. We transformed the numerical data into dichotomous data that is 1for fall risk, and 0 for no fall risk to check the validity of the tested variables in binary regression model. The equation model in which Odd Ratio that is probability of (A) P (B) in which we interpreted that which independent variable is significantly affect falls or not falls. The beta coefficient values (Exp.b) greater than 1 were considered as the cutoff point to indicate a higher probability of the selected outcome occurrence, whether it is falls or no falls, in the model in relation to the predictive variables [17].

#### Validity of the proposed model

The validation research was conducted to evaluate the discriminant validity of the model. This was done by plotting ROC curves for 30% of the subsamples. By analyzing the differences between the areas under the two curves, the calibration validity of a model was established [23]. It is necessary to ascertain whether the predicted probability obtained for subsample, and using the models created agrees with the observed probabilities of model 2.

## Results

The demographic features, cognitive status, and TUG level of the participants were assessed. The average age of the study participants was 68.1 + 5 years (ranging from 60 to 82 years old). When education level is taken into account, 42% of the participants were illiterate, and only 14% are recorded as graduates; more than half (54% of the participants in this study were female). Cardiovascular diseases were common (31%), the majority (52% were living with join family, and most of them were financially dependent on other family members, which is 38%. More than half, or 51%, of the study participants reported at least one fall in the last year. Body movements in five time prayers accounted for 32% of those who did not follow all the due to health issues or personal preference. The cognitive level of the study participants was 47% moderately affected, and only 53% had active memory in this study. Older adults in this study had poor vision, as 66% reported having a visual problem.

### Falls reports

The majority of the participants were belong to Pathan tribe that is (40%) in this study. Female participants (44%), as well as those aged 65 to 70 years, reported more falls. In this study, 42% of the participants were illiterate. Additionally, 59% reported having a living spouse, while the same percentage had an additional source of income. Furthermore, 56% of the participants lived in an extended family system. The study also found that 93% of the participants had a joint problem, 75% had a visual problem, and 71% did not follow body movements during prayer. (see Table 1).

**Table 1:** Demographic characteristics, fall frequency, and risk factors of fall occurrence

Demographic Variables.*N* = 140		Total#	Fall history		*P. Value*
		**%**	**yes%**	**No %**	
Gender	Male	46%	46%	54%	0.49*
	Female	54%	44%	56%	
Age	60–65	46.40%	33%	67%	0.04*
	66–70	14.30%	44%	56%	
	71–75	4.30%	16%	84%	
	> 76	8.57%	33%	67%	
Education level	Graduate	14%	14%	86%	0.021**
	Matric Secondary	22%	22%	78%	
	Matric Secondary	17%	20%	80%	
	Illiterate	47%	42%	58%	
Married	Alive	79%	59%	41%	0.012**
	Widow	21%	41%	59%	
Source of Income	Pension	28%	57%	43%	0.62
	Others	34%	59%	41%	
	No, any source	38%	48%	52%	
Health status	Cardiovascular	30.70%	51%	49%	0.72
	Diabetic	22.00%	42%	58%	
	Joint Pain	20%	93%	7%	
	Other	4.30%	33%	67%	
	No diseases	9.30%	46%	54%	
Living status	Join family	47%	54%	46%	0.45
	Extended Family	52%	56%	44%	
Visual problem	yes	52%	75%	25%	0.002**
	No	47%	33%	67%	
Prayer practice	five-time as prescribe	48%	37%	63%	0.000***
	Not follow seven steps	52%	71%	29%	
Ethnicity	Pathan	0.4	58%	42%	
	Panjabi	0.3	47%	53%	
	Sindhi	0.2	40%	60%	
	Chitrali 8%	0.1	21%	54%	
Cognitive level	Normal	54%	45 64%	55%	0.000***
	Moderate	47%		36%	
	< 12 s	48%	31%	69%	
TUG level	13–19 s	28%	64%	56%	0.001**
	> 20 s	24%	90%	10%	

In the Table 1 TUG=Time Up and Go, P. value <0.000^***^ highly significant relationship, P. value, 0.02 indicates moderately significant, and P. value <0.05^*^ indicates low level of significant relationship between dependent and independent variables

### Factors associated with falls occurrence

There was a significant relationship be-tween income level and education, health, and age as scores ranged from > 70 to 0.90. Visual equity and age are also correlated with scores > 0.70. In our study, health, status, gender, and living status did not show any significant association (*P* > 0.005) in all the mentioned variables. The study found a significant association between moderately low cognitive status, poor vision, and a history of falls (*p* < 0.001). Visual health and age are also correlated with scores greater than 0.70%. In our study, we found no significant association (*P* > 0.005) between health, status, gender, and living status in all the variables mentioned. The significant value of moderately low cognitive status and poor vision was associated with a fall history (P.0.001). In Table 1, the frequency of falls is higher in females compared to males. The age group of 66 to 70 years reported the highest number of falls, with the data presented as percentages in the descriptive statistics. Among female participants, 44% reported falls, while the age group of 65 to 70 years reported a history of falls in 43% of cases in this study.

### Binary logistic regression model

**Fig. 2:**
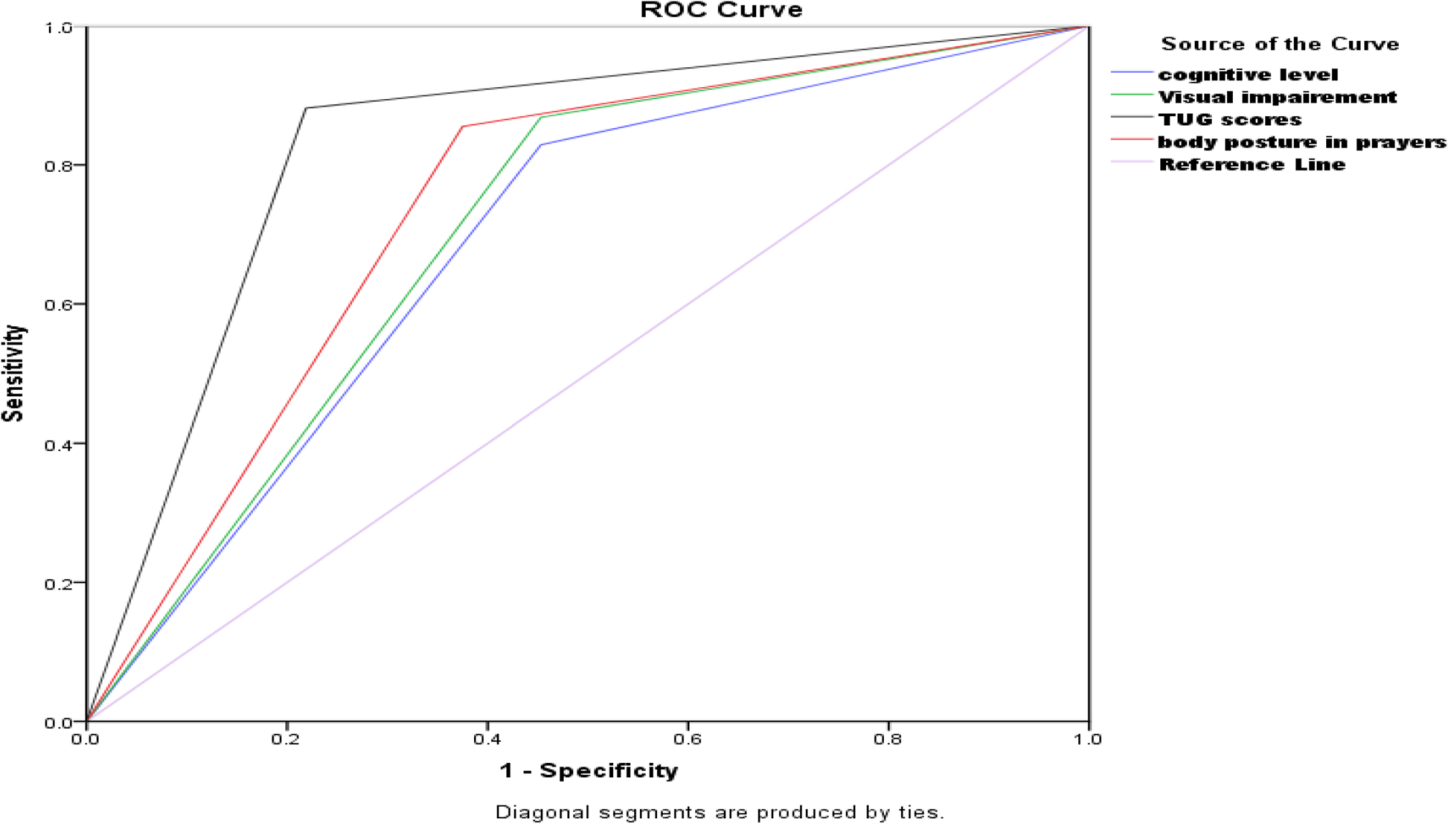
Receiver Operating Characteristic (ROC) curve of the four predictive variables

The goodness of the model is statistically significant (*p* < 0.001). Age, gender, economic status, education status, health status, and ethnicity were removed from the model as they were found to be non-significant (*P* > 0.005) in both the Omnibus Chi-square test and the Hosmer and Lemeshow test. TUG level, cognitive status, poor visual equity, and proper body movement in prayers were good fits of the model, as the chi-square test values of each variable in the area model were statistically significant at (P.0.05) and 94% in the Hosmer test. The variation in the criterion variable is significant, as the pseudo-R square of the approximate variation in the criterion variable through the adjusted versions of Cox and Snell R and Nagel-kerke R shows a 77% change in the fall risk can be accounted for by the predictive variables in this study. In the Table 2, our model correctly predicted that fall risk using 81.3% of the selected variables, and the Fig. 2, the AUC in the ROC shows the high sensitivity and specificity of our hypothetical testing variables in the relationship with fall risk in this study.

**Table 2:** Model of fit analysis under Binary Logistic regression analysis of predictive variables

Omnibus Tests of Model Coefficients		Chi-square	df		***p. value***
		65.961	4		0.000
		65.961	4		0.00
		65.961	4		0.000
Hosmer and Lemeshow Test	likelihood	4.071	7		0.772
-2 Log likelihood	115.302^a^	Cox & Snell R Square	0.526	Nagelkerke R Square	0.77
**Classification Table**		fall risk Predicted	Percentage of model correctness		
		No = 0	Yes = 1	%	
Fall risk	0	60	16	78.9 specificity	Overall 80%
	1	12	58	81.3 sensitivity	

Table 2 represent the overall 80% model fit index in the classification table and the Negelkert value is > 0.50 indicated that the four predictive variable are significantly associated with fall occurrence

### Receiver operator characteristic curve

The receiver operating characteristic curve was generated for all the variables to discriminate older adults with falls and no falls. The total area under the curve is representing the sensitivity, and specificity of the tested variables. The study found that lack of body movement during prayers covered 82% of the total area, poor cognitive status covered 80% of the area with a 95% confidence interval, poor vision accounted for more than 85% of the area, and a TUG level greater than 85% accurately predicted fall risk in the study participants. These factors were strongly associated with fall risk in the participants. The area under the curve (AUC) of this study model is shown in graphical lines of sensitivity and specificity of the test variables in which all the lines of four variables are moving from left to right with the top point of > 80% which is near 1. The model is 80% correctly predicting that variables are correctly predicting falls risk in older adults. (See Fig. 2)

In Fig. 2, the model > 80 percent accurately classify positive cases, providing insights into its discriminatory power and reliability. The curve's shape and the area under the curve (AUC) values showed > 80% in each highlight the model's effectiveness in distinguishing between positive and negative instances.

### Model predictive value

**Fig. 3:**
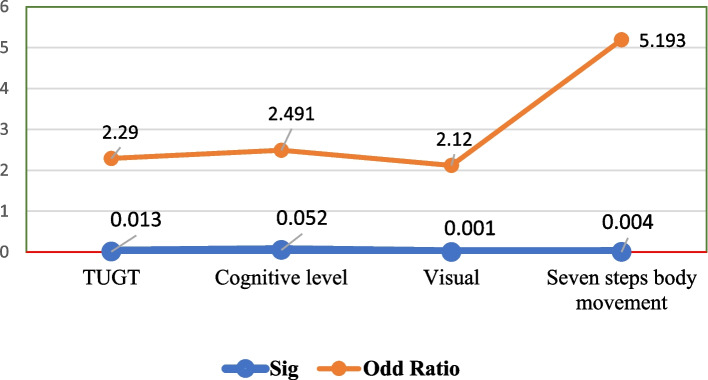
Odd Ratio of fall risk with hypothetical variables

To determine the predictive values in our model, we examined the equation model where the beta coefficient Exp (b) value is greater than 1, indicating a higher likelihood of the chosen outcome happening. Three independent variables, namely cognitive function, TUG level, and poor vision, were found to predict fall risk in older adults in this study. The log odds of TUG falling are 2.29 times higher when the Exp (b) value is within the range of L = 1.11 and U = 439.78. Older adults' cognitive level is slightly lower than expected to predict the occurrence of falls with a Beta value of B (2.49) in Exp. B (95%) with a value of 0.052, lower at 0.65, and upper at 4.59, but it still needs to be considered in future studies to avoid neglecting this factor. The body movements in prayers predicted the occurrence of falls five times more than other factors such as poor vision, cognitive health, and walking speed (*p* = 0.004, Exp (b) = 5.19). In our prediction model, the vision scores are highly significant for the probability of fall occurrence (*p* < 0.001). The odds ratio with hypothetical variables in Table 3 and Fig. 3 showed that the model is the best fit in the Omnibus test (*P* < 0.005). The Hosmer & Lemeshow likelihood value, greater than 0.50 (0.77 in Nagelkerke R square), indicates that our model has a good fit with an overall validity and reliability score of 80%. It indicates that the four predictive factors are not identified by chance. This result indicates that poor vision, lack of physical movement, poor gait, and walking ability in the TUG 3-m walking test for 30 s, as well as low cognition on a scale of 10 points, demonstrate the significance of the model's appropriateness and its potential adoption in future fall risk assessments. Figure 2 shows that the model predicts the probability of falling based on the tested variables in this study, represented as the Odds Ratio (Exp(b)). All variables in the Equation model have an Odds Ratio greater than 1, indicating their significance in predicting falls. Specifically, the Odds Ratios for TUG, Cognitive scores, Visual, and not moving the body during prayer are 2.29, 2.4, 2.1, and 5.1, respectively. When the score is less than 1, it means that the model does not indicate any probability of falls in the future.

**Table 3:** Odd Ratio of fall risk with the relationship of the predictive variables such as TUG, cognitive level, Poor vision, and prayer method in the Equation model. *N* = 140

Risk factors of falls	B	S.E	Wald	Sig	Exp(b)	95%C.I.for EXP(B)
**Step 1**^**a**^ TAGS level	0.828	0.333	6.196	.013*	2.29	1.193 4.396
Cognitive level	0.913	0.47	3.778	0.052	2.491	1.992 6.255
visual	2.112	0.552	14.626	.000*	2.121	0.041 0.357
Body movement in Prayers	1.647	0.576	8.191	.004*	5.193	1.681 16.043

Table 3 presents odds ratios associated with fall risk and predictive variables such as TUG, Cognitive Level, Poor Vision, and Prayer method in the Equation Model, providing coefficient values (B), standard errors (S.E.), Wald statistic, and significance levels (*P*. value). Odds ratios (Exp (b)) are offered, along with 95% confidence intervals (95% C.I. for Exp (B)) for each predictive variable. The significance level is denoted by ^*^ at *p* < 0.05.

In Fig. 3, the Odd ratio indicated the true relationship in this predictive model, and it provides insights into the influence of four independent variables on the likelihood of fall occurrence.

### Validity of the Model

**Fig. 4:**
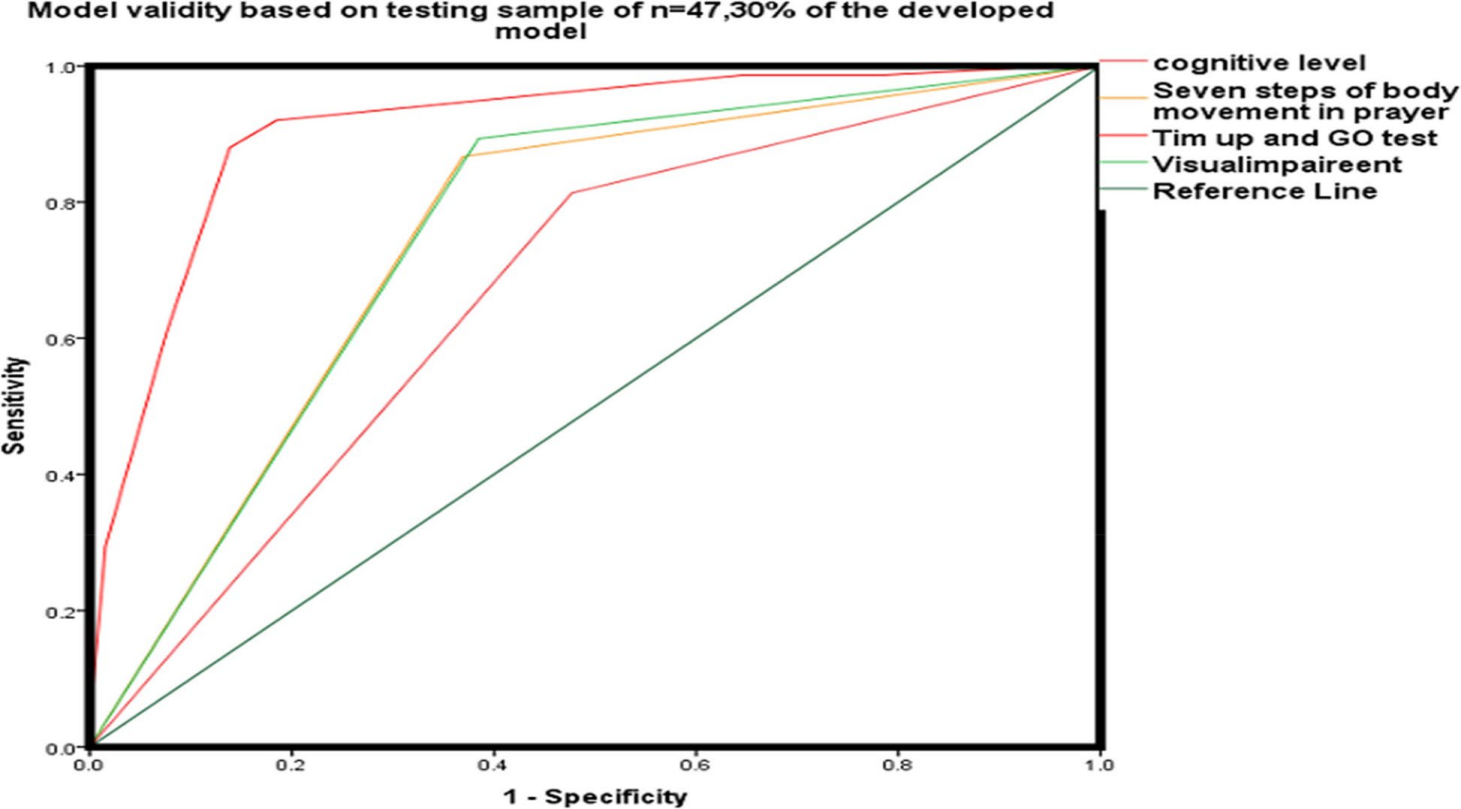
The test Result variable(s) AUC of cognitive level 75%,seven step body movements 80%, TUGT 90% and visual impairment was 75%.All the tested variables showed at least one tie between positive actual state and negative state group

The AUC in the ROC of the randomly selected testing sample from the proposed model. The AUC of the testing sample in Fig. 4 supports that this model is > 80% valid and reliable, and the chance of overfitting is not occurring in the model. The AUC in ROC is greater than 70% of the area above the straight line, indicating that this model is not over-predicting the fall risk factors in older adults. The risk factors are not attributed to chance. We are confident in supporting the correctness of our model.

Fig 4 Validity of the Model AUC in the ROC of the randomly selected testing sample from the proposed model

The ROC figure shows an AUC of the testing sample, indicating that this model is valid with an accuracy of over 80%. Therefore, the chance of overfitting in the model is not high. We are confident in our model's ability to assess falls risk in elderly individuals of Pakistan who share the same ethnicity and geographical location. The AUC in ROC is greater than 70% of the area above the straight line, indicating that this model is not over predicting the fall risk in older adults by chance. The odds ratio of the testing sample is greater than 1 in the graph, indicating a high probability of fall occurrence in the study population. The significant value of exercise's four independent variables is significant predictors (*P* < 0.005), as displayed in Fig. 4. This figure shows the external validity of the model. We randomly selected 30% of the sample as a testing sample, while the remaining 70% was considered a training sample. The binary logistic regression shows that the exponentiated beta (Exp.b) for each variable is greater than 1, indicating an odds ratio greater than 1.

### The external validity

**Fig. 5:**
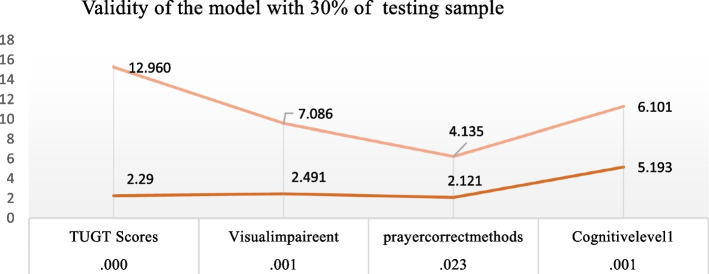
Revealed distinct influence patterns for the four variables. The odds ratio of the entire model indicates the highest odds Ratio of fall risk with a unit increase in TUGT score, a unit decrease in cognitive function increases fall risk. Poor vision yielded an odds ratio of 7.086 (95% CI), implying over double the odds of falling for individuals with poor vision. The odds ratio for the prayer method was 4.135 (95% CI), which indicates a moderate association with fall risk and is significantly linked with fall risk with *P*. value of 0.02

The external validity was assessed by using randomly selected data from the model. We divided our data into two groups: 70% for training data and 30% for testing data. These groups were created from the 140 samples collected using a random sampling method. The significance of the test in Fig. 5 indicates that the test is significant (*P* < 0.005) for all four predictive factors of fall occurrence, and these factors did not occur by chance in this study.

Figure 5, Validity of the model with Odd Ration with sample of 30% of the total

## Discussion

Our purpose in this study was to identify risk factors associated with falls among older adults living in the community in Pakistan. Additionally, we aimed to develop a predictive model to assess fall risk in healthy older adults who are at risk for disability. We examined various fall risk assessment tools, including the TUG checklist, 10-point cognitive screening (10-CS) scores, seven steps of body postures in Muslim prayers with (1–7) scores, visual equity scores, and fall history in the last six months. In the demographic data, the average age of the study participants was 68.1 years, ranging from 60 to 82 years, which is similar to the previous study conducted in Faisalabad, Pakistan [2]. The difference in the age distribution did not come as a surprise to us. This is because older adults, specifically those under the age of 60 to 65, generally have a lower risk of falling. This can be attributed to their engagement in physical activity. Additionally, this age range represents a transitional period from adulthood to older adulthood. Similarly, older adults over the age of 75 mostly become dependent on others for daily living activities due to deteriorating health conditions and disabilities, which limit their ability to participate in this study [7, 40]. When education level, it was found that 42% were illiterate, while only 14% were recorded as graduates. Additionally, more than half, or 54%, of the participants in this study were female. Our results are inconsistent with studies conducted in other countries where older adults [30, 41]. A meta-analysis study conducted by Wiednmann et al. found that training older adults with low literacy on daily exercises, safety measures, and strategies to avoid fall-inducing situations might help reduce the risk of falls. Education was identified as one of the significant variables that had a negative impact on fall risk prediction in the previous study [23]. We believe that education provides older adults with opportunities to live a safe and secure life in old age [42].

### Prevalence of fall occurrence

The frequency of falls among the study participants in this study was 55%, which is relatively high compared to previous studies. For instance, a study conducted in Pakistan reported a fall frequency of 44% [2]. However, the frequency of falling was low, with only 18% of falls reported in the Chinese older adult population [43, 44]. The difference may be due to a lack of regular exercise, cultural values, and advancements in gerontology research aimed at improving the quality of life in older adults. On the other hand, our results were quite similar to a study conducted in Taiwan, where the frequency of falls among community-dwelling older adults was reported to be 33% [45]. In our study, the frequency of falls was high among individuals aged 65 to 70 years, which contrasts with a Brazilian study where adults over 75 years old reported more falls [4]. This can be attributed to several factors, including the absence of risk assessment strategies, inadequate physical exercise, low literacy rates, and a lack of awareness programs promoting healthy aging in Pakistan [8, 46].

### Associated factors of fall occurrence

We conducted an analysis to forecast the fall risk and identify risk factors for falls in older adults. Some of the highlighted risk factors in our study were slow walking speed, impaired cognitive health, lack of body movements during five-time prayers, and impaired vision. Other factors associated with falls in our study participants included being between the ages of 65 and 70, illiteracy, and being female. These risk factors were found to be correlated with fall experiences (*P* < 0.005). Our findings were contradicted by a study conducted in the United States of America, which identified medication use and fear of falling as more prominent risk factors for falls in older adults [17, 47]. Similar risk factors have not been observed in other studies due to the multifactorial nature of falls and the predominantly contextual nature of these factors. However, in our study, the population belonged to the Muslim community, with a majority being females who stay at home and have limited opportunities for physical activities. However, they do have the opportunity to engage in physical movements five times a day through distinct body postures [2, 8]. Lack of prescribed body movements during the five times of prayer was one of the prominent factors contributing to the risk of falling in this study. Complaints of poor vision, slow walking speed, and low cognitive ability were significantly correlated with a history of falls (*p* < 0.005). The previous study identified the same factors, except for the lack of body movement during prayers. This indicates a sedentary lifestyle and a lack of physical activity in older adults, which is added as a new predictive factor significantly correlated (*P* < 0.04) with fall occurrence in this study [24]. The three factors, namely poor vision, low cognitive level, and a poor score in walking speed, were consistent with another study conducted by Makino et al. In their study, they also emphasized poor cognition and visual problems as factors contributing to falls in older adults [40]. Poor gait and balance problems increase the risk of falls, which can be assessed using various tools such as the Timed Up and Go Test (TUGT) and the Short Physical Performance Battery (SPPB), among others. The TUGT is a frequently used tool and a strong predictor of adverse outcomes. It provides a range of reference values that depend on different age groups and associated factors [48]. TUG scores remained higher than 30 s for stroke survivors compared to healthy older adults who completed the 3 m walk test in less than 20 s in the previous study [49]. Our study also found that completing a 3-m walk in more than 19 s during the Timed Up and Go Test (TUGT) increases the likelihood of future falls in healthy older adults (*p* < 0.005). The cutoff point of TUGT test time in our study was < 19 s, indicating a low risk of falling. However, this finding is not consistent with another study, which reported TUGT times of 60 s, 70 s, and 80 s as 7.91 s, (6.62, 9.20), 8.67 s (7.23, 10.12), and 11.68 s (8.11, 15.26) with a 95% confidence interval, respectively. Some sociodemographic variables correlated with the occurrence of falls in this study exhibit similarities to those observed in the Brazilian study [5]. In both studies, sociodemographic characteristics, vision impairment, and cognitive impairment were found to be associated with an increased risk of falling. However, surprisingly, the age group of > 65–70 years old was identified as a higher risk group for falling in one study, which contradicts our findings. Gender, specifically being female, was found to be significantly associated with a higher risk for falls (*p* < 0.05) in our study. This finding is consistent with a previous Brazilian study, which also reported that females experienced a higher occurrence of falls [50]. Female participants were found to be at a higher risk in another study conducted by Khatak et al., where females accounted for 32% of the total fall prevalence [30]. According to other research, women have a higher tendency to fall than men do. This difference can be attributed to women's physiological differences in bone and muscle structure, menopausal hormone changes, and multitasking [44, 51]. In Pakistan, there is a male-dominated culture where females have limited opportunities to participate in physical activities. This study identified several protective factors associated with a lower risk of falls. These factors include being male, having a high level of literacy with a graduation degree, and performing proper body movements during five-time prayers with prescribed body motion. These factors were found to be significantly associated with the non-fallers group (*P* < 0.05). The finding was consistent with the study conducted by Adriana et al., in which education, gender, and social support were identified as protective factors in preventing falls among older adults [5]. Poor gait was significantly correlated with fall risk (*p* < 0.05) in our study. The same findings were observed in a study conducted in the USA on 92 older adults. The study found a strong correlation between poor scores in cognitive status, medication-related drowsiness, poor gait, and balance, and the occurrence of falls [26, 31]. Although this study has focused on sociodemographic, contextual, intrinsic, and behavioral factors of falls and gait speed assessment. The importance of performing five-time prayers should also be emphasized, as there is a likelihood of these events occurring due to a lack of physical activity, particularly among Pakistani women. Hence, this model might serve as a valuable tool for differentiating between elderly individuals residing in Muslim communities in Pakistan who are at a high risk of falling and those who are not at risk. Healthcare professionals could utilize our model to assess and screen the identified risk factors in the general population. This would enable them to take the necessary actions to prevent fall risks in older adults in Pakistan.

Lack of physical activity and exercise increases the vulnerability to falls and disabilities in older adults [30]. According to a systematic review study, engaging in the practice of a rhythmic slow-motion physical exercises has the potential to enhance the overall physical and psychological health of older adults [52]. The seven-step body movements in Muslim Salat constitute a standardized, rhythmic, and slow-motion exercise, closely resembling other rhythmic exercises [53]. It involves little effort with high concentration (standing, bowing, prostration, and sitting) to improve joint flexibility, muscular strength, balance, and body circulation when practiced regularly in old age [54].

The present study investigated the influence of seven-step body movements performed during Muslim Salat on the risk of falls among older adults. Our discussion highlights the significant relationship observed between the level of body movements and the risk of falls occurrence, with a cutoff point of at least five movements out of a total of seven. Adhering to prescribed body movements led to a notable reduction in fall incidents. This suggests that integrating these motions into the daily routines of healthy or pre-disabled older adults positively influences fall reduction and prevents disabilities in old age. These movements encompass several mechanisms consisting of transitions and weight shifts, as these movements are very gentle and with less side effects [18, 24, 33, 54]. They effectively engage various muscle groups and coordination abilities while promoting joint mobility, flexibility, balance, and postural control specifically during bowing and prostrations,. Noncompliance can increase the risk of falling. This study's alignment with existing research, particularly in yoga and tai chi [16, 55, 56], reinforces the role of body movement in reducing fall risks. Furthermore, the prescription of each bodily movement within the Muslim prayer depends on a prior risk assessment, aiming to reduce the potential for falls and injuries in older adults. Therefore, prior to recommending all seven steps for practice in old age, it is imperative to provide precise training, guiding booklets, or video clips to ensure accurate independent practice. Literature suggests that conducting a risk assessment is crucial before prescribing exercises for an individual. Exercise steps can be modified based on the difficulty of performance. If an individual finds it very difficult to perform certain exercises, they should not be prescribed [1]. However, a previous study reported that the seven steps of Muslim prayers could improve physical function, mobility, and balance in healthy or pre-frail older adults. These exercises are considered safe and low-risk [54].

### Validity of the model

Our model is a good fit due to its internal validity. The Nagelkerke R Square ranges from 52 to 77%, which strongly supports the prediction that variables such as poor cognitive level (scores > 7 on a 10-point scale), poor vision, gait problems (taking > 19 s to walk a 3-m distance), and lack of seven-style body movement in prayers are significant factors contributing to the risk of falling. This model supports the idea that physical activity is an important indicator for preventing falls in older adults. The odds ratio is greater than 1 in the AUC model shown in Fig. 4. The coefficient Ex (b) of the independent variables shows a value greater than 1 in each predictive variable, such as TUGT (2.290), cognitive health (2.491), visual health (2.121), and prayer body movements (5.193). In terms of the components of our model, previous studies have shown a correlation between the fall histories of older individuals, low cognitive scores, TUG scores, pain levels, and age group with falls in older adults [32]. Hence, the items we selected for our binary regression model analysis corroborated this finding with different models, such as the simplified decision-tree algorithm model [40], and the Shapley Additive Explanations model [23]. The binary regression model is a logistic regression model [57] that can be used to reveal stratified relationships between fall predictors and subsequent fall risk. It includes TUG (Timed Up and Go test), cognitive problems, lack of body movement during prayers, and poor visual equity in an importance-based ordering of predictors. TUG is located at the top of the ROC graph. Poor gait speed is the most influential predictor and is recommended to be assessed to predict falls in the future. Therefore, we believe that the structure of our model is valid and reliable to assess fall risk among older adults in Pakistan. Additionally, our model calculated the optimal cut-off point of cognitive level as seven out of 10 scores for fall prediction. Regarding the cut-off point of TUG for falls, a systematic review study [27], mentioned an average time of 10 to 32 s for both individuals with a history of falls and non-fallers. Additionally, a cutoff point of < 19 s was mentioned to measure physical mobility in healthy older adults [30]. Our cut-off point was 19 secs, which is relatively more power to include all healthy older adults. The binary regression model outperformed the logistic regression model in terms of AUC, accuracy, sensitivity, probability, and odds ratio, making it suitable for use as a primary screening tool for fall risk. In the figure, the testing model is shown to be more than 70% accurate.5 indicated that the primary model was a valid model as the odd ratio Exp (b) of each variable is > 2, with (*P*.0.01) which supports that our model is valid [35]. The fall risk model can provide guidance to healthcare providers, allowing them to focus on specific factors of fall risk instead of analyzing individual assessments.

## Conclusions

Falling is multifactorial; we cannot attribute falls in older adults to a single factor. To provide information on the prevention and identification of seniors at risk, multidisciplinary expertise is necessary. This justifies the need for conducting this study. Assessment of visual health, cognitive impairment, gait speed, and the seven steps in prayer can be selected as fall risk assessments for Muslim older adults, as these variables are linked to the probability of falls in the binary logistic regression model. Therefore, this study could help community health nurses and public health workers incorporate these predictors into their fall risk assessment tools. This would enable them to provide suitable fall prevention strategies for healthy Muslim older adults.

### Study limitations

The study does have certain limitations. The data is retrospective; therefore, in the future, conducting more comprehensive analyses with additional predictor variables and prospective studies could potentially enhance the results. However, these findings may not be applicable to other older adult populations. To validate the threshold values of the variables in our model, further longitudinal studies are necessary. A fall risk assessment checklist, the Berg Balance Test, certain medications, a sleep quality assessment, and nutritional status can be included in future studies.

### Future implications

This study's model was constructed to assess fall risk among older adults through cognitive assessment, visual assessment, seven steps of prayer body movements, and the Time Up and Go test, which can be further, examined in randomized control trials. While we think this can be applied successfully to forecast fall risk in Peshawar and in other Muslim populations that are similar, Our study provides valuable information on future predictors; therefore, selecting factors for the prediction model, a literature review, and clinical experience are often used to prevent bias in predictor selection. It is essential to examine the external validity of the developed model in the general population in order to assess the model's stability across different populations.

## Data Availability

The data set used and/ or analyzed during the current study is available from Dr. R.B can be provide as per request.

## Abbreviations

WHO: World health organization
CDC: Center of chronic diseases
EPHPP: Effective public health practice project
PICO: Population, intervention, control group, outcome
RCT: Area under curve
ROC: Receiver operator characteristics
ADL: Activity daily living
TUG: Time Up and Go
AUC: Area under Curve
DHO: District Health Officer
ZZU: Zhengzhou University
IRB: Institution of ethical review board
BHU: Basic health unit
SPSS: Statistical package of social sciences
MMSE: Mini mental status examination
