# Correction: Assessment of fall-associated risk factors in the Muslim community-dwelling older adults of Peshawar, Khyber Pakhtunkhwa, Pakistan

**DOI:** 10.1186/s12877-023-04486-w

**Published:** 2024-01-02

**Authors:** Rashida Bibi, Zhang Yan, Muhammad Ilyas, Mussarat Shaheen, Satya Narayan Singh, Akhter Zeb

**Affiliations:** 1 Institution of Nursing and Health Sciences, Zhengzhou University, Zhengzhou, Henan China; 2 School of Nursing, Iqra National University, Peshawar, Khyber Pakhtunkhwa Pakistan; 3 Government Nursing College Abbottabad, Khyber Pakhtunkhwa, Pakistan; 4 Torrens University, Adelaide, South Australia Australia; 5 Ismail College of Nursing Sawat, Khyber Pakhtunkhwa, Pakistan

**BMC Geriatrics (2023) 23:623**

10.1186/s12877-023-04322-1

After publication of this article [1], the authors reported that in this article the affiliation details for the 4th author were incorrectly given as ‘Government College of Abbottabad, Khyber Pakhtunkhwa, Pakistan’, but should have been ‘Government Nursing College Abbottabad, Khyber Pakhtunkhwa, Pakistan’.

The original article [1] has been corrected.
